# Health Tract, No. 69

**Published:** 1862-05

**Authors:** 


### HEALTH TRACT, No. 69.

### READ AND HEED.

"The great object of labor is profit; and to attain that, system and care are indispensable. Therefore,

1. Be Punctual.—Every man has a right to demand his salary to the last cent, and the employer a right to the time agreed upon to the last minute. The man who fails to fulfill his agreement in this particular, is no less dishonest than he who gives short change, weight, or measure.

2. Be Industrious.—You have been engaged to do all you can during ten hours of each day; and your employer has a right to a hearty, ungrudged service. If your regular work is for a time stopped, look for something else to do; keep steadily and regularly busy.

3. Be Careful.—Never lose sight of the fact, that the employer is as dependent for a living upon his profits as you are upon your salary. Every loss of type, paper, plates, ink, or time, caused by carelessness or waste, amounts, practically, to a reduction of his salary, without his consent.

4. Be Quiet.—This large building was erected for work, not play. If you have to wait for any thing, do so quietly. Damage is sure to result from 'sky-larking,' either to type, cases, forms, paper, plates, presses, or yourself; for an habitual trifler, or noisy person, will certainly be discharged.

5. Be Gentle in Speech.—Swearing will not correct an error, mend a batter, clean a roller, or make a press work well; patience and oil will do much more, and be every way better. Cursing may make your fellow-workmen angry, and prevent them helping you, when gentle words would have commanded ready help.

6. Be Clean.—If you like to be dirty yourself, you can not be allowed to inconvenience your fellow-workmen, soil the office, and spoil the work, by habitual filthiness. Soap, lye, water, and towels have been provided; so there is no reason why you should not be clean. It is easier to keep clean than to make clean.

7. Be Obedient.—Those who have direction of your work have responsibilities you know nothing of. Even if they should be harsh or abrupt in their commands, nothing can be allowed to excuse disobedience. Obey, or leave the office, must be the inevitable rule. The employer will judge fairly of any complaint of an unjust command, but not until it has been obeyed. In short,

8. Be Just—To yourself, your fellow-workmen, and your employer; and in the intelligent application of this rule you will find and give perfect satisfaction, and prove that cleanliness, honesty, industry, obedience, and carefulness for others' feelings, mark the demeanor of a printer, or pressman, not less than that of a gentleman.

The acceptance of a situation or of temporary employment in this office, will be regarded as a pledge of obedience to these rules."—D. M. Cole.

But while a careful, systematic, and just industry will uniformly be attended with pecuniary success, the more ultimate object of life, a quiet, peaceful and happy old age, can never be attained without bodily health.

To that end
We append
Eight Cs
To as many Bs.

1. See to it that you eat regularly, slowly, temperately.
2. See to it that you sleep early and abundantly.
3. See to it that cold water is your great stand-by beverage.
4. See to it that you never work to exhaustion.
5. See to it that the bodily habits are as regular as the day.
6. See to it that you never work or rest in chilliness.
7. See to it that you cool off very slowly after all forms of exercise.
8. See to it that you place your head upon your pillow every night with a view to speedy, sound connected and invigorating sleep, with "a conscience void of offense toward God and toward men;" and strive steadfastly and heroically to maintain the same, from the early morning of each succeeding day. Thus you will infallibly, because God has said it, secure the fruition, as well as "the promise of the life that now is, and of that which is to come." A reward how ample!—how magnificent!—in quality glorious; in extent infinite!

